# Benfotiamine, a Lipid-Soluble Analog of Vitamin B_1_, Improves the Mitochondrial Biogenesis and Function in Blunt Snout Bream (*Megalobrama amblycephala*) Fed High-Carbohydrate Diets by Promoting the AMPK/PGC-1β/NRF-1 Axis

**DOI:** 10.3389/fphys.2018.01079

**Published:** 2018-09-03

**Authors:** Chao Xu, Wen-Bin Liu, Ding-Dong Zhang, Hua-Juan Shi, Li Zhang, Xiang-Fei Li

**Affiliations:** Key Laboratory of Aquatic Nutrition and Feed Science of Jiangsu Province, College of Animal Science and Technology, Nanjing Agricultural University, Nanjing, China

**Keywords:** benfotiamine, glucose metabolism, mitochondrial biogenesis, mitochondrial function, *Megalobrama amblycephala*

## Abstract

This study evaluated the effects of benfotiamine on the growth performance and mitochondrial biogenesis and function in *Megalobrama amblycephala* fed high-carbohydrate (HC) diets. The fish (45.25 ± 0.34 g) were randomly fed six diets: the control diet (30% carbohydrate, C), the HC diet (43% carbohydrate), and the HC diet supplemented with different benfotiamine levels (0.7125 (HCB1), 1.425 (HCB2), 2.85 (HCB3), and 5.7 (HCB4) mg/kg) for 12 weeks. High-carbohydrate levels remarkably decreased the weight gain rate (WGR), specific growth rate (SGR), relative feed intake (RFI), feed conversion ratio (FCR), *p*-adenosine monophosphate (AMP)-activated protein kinase (AMPK)α/*t*-AMPKα ratio, peroxisome proliferator-activated receptor-γ coactivator-1β (PGC-1β) and nuclear respiratory factor-1 (NRF-1) protein expression, complexes I, III, and IV activities, and hepatic transcriptions of cytochrome b (CYT-b) and cytochrome c oxidase-2 (COX-2), whereas the opposite was true for plasma glucose, glycated serum protein, advanced glycation end product and insulin levels, tissue glycogen and lipid contents, hepatic adenosine triphosphate (ATP) and AMP contents and ATP/AMP ratio, complexes V activities, and the expressions of AMPKα-2, PGC-1β, NRF-1, mitochondrial transcription factor A (TFAM), mitofusin-1 (Mfn-1), optic atrophy-1 (Opa-1), dynamin-related protein-1 (Drp-1), fission-1 (Fis-1), mitochondrial fission factor (Mff), and ATP synthase-6 (ATP-6). As with benfotiamine supplementation, the HCB2 diet remarkably increased WGR, SGR, tissue glycogen and lipid contents, AMP content, *p*-AMPKα/*t*-AMPKα ratio, PGC-1β and NRF-1 levels, complexes I, III, IV, and V activities, and hepatic transcriptions of AMPKα-2, PGC-1β, NRF-1, TFAM, Mfn-1, Opa-1, CYT-b, COX-2, and ATP-6, while the opposite was true for the remaining indicators. Overall, 1.425 mg/kg benfotiamine improved the growth performance and mitochondrial biogenesis and function in fish fed HC diets by the activation of the AMPK/PGC-1β/NRF-1 axis and the upregulation of the activities and transcriptions of mitochondrial complexes as well as the enhancement of mitochondrial fusion coupled with the depression of mitochondrial fission.

## Introduction

As the most economical energy source, carbohydrates are now being commonly incorporated into aquafeeds to improve the physical quality of the feed and reduce the catabolism of proteins and lipids by aquatic animals (Yang et al., 2018). However, it is generally acknowledged that fish show poor capability in utilizing carbohydrates for energy purposes than terrestrial animals (Hemre et al., 2002; Enes et al., 2009). Furthermore, most species (especially carnivorous ones) often exhibit prolonged hyperglycemia after an intake of carbohydrate-enriched diets or a glucose load (Moon, 2001; Kamalam et al., 2017) as is similar to the symptoms of type 2 diabetes mellitus observed in mammals. Previous studies have suggested that the poor carbohydrate utilization or the persistent hyperglycemia in fish may be attributed to the relatively low number of insulin receptors, the low affinity of glucose transporter proteins for glucose, a poor inhibition of postprandial gluconeogenesis, poor hepatic lipogenesis from glucose, etc (Panserat et al., 2000; Enes et al., 2009). Recently, several approaches such as metabolomics and transcriptomics are being employed to assess diet-induced metabolic syndromes in fish (Miao et al., 2017; Prathomya et al., 2017; Prisingkorn et al., 2017). Findings from such studies suggest that the disruption of the energy homeostasis in fish is closely implicated in the development of disturbances in the glucose metabolism. Therefore, further biochemical and molecular investigations of the energy metabolism are necessary and will undoubtedly facilitate better understanding of the utilization of carbohydrates by fish.

In eukaryotes, the mitochondria are regarded as the most important organelles responsible for cellular ATP synthesis and metabolism (Scheffler, 2008). Mitochondrial function mainly depends on the mitochondrial content and shape (Tang et al., 2014). Generally, increased mitochondrial content has been observed in response to challenges involving high-energy demands, such as in electrical stimulation, exercise, cold, heat, stress, etc. (Booth and Thomason, 1991; Cannon and Nedergaard, 2004; Bremer and Moyes, 2011; Gao and Moyes, 2016). Mitochondrial biogenesis is a complex and precise process involving the replication of mitochondrial DNA (mtDNA) and the expression of nuclear and mitochondrial genes (Tang et al., 2014). In this regard, adenosine monophosphate (AMP)-activated protein kinase (AMPK), known as the cellular “fuel gauge,” may play a central role, since the energy homeostasis mediated by it is closely related to the biosynthesis and function of mitochondria (Zhao et al., 2013). Also, the AMPK can be activated by a variety of metabolic stresses that typically increase the cellular AMP/ATP ratio, such as hypoxia, exercise, a glucose load, energy restriction, and so on (Hardie et al., 2003). Once activated, AMPK phosphorylates the mitochondrial master regulator: peroxisome proliferator-activated receptor γ coactivator-1 (especially PGC-1α and PGC-1β isoforms) (Reznick and Shulman, 2006; Bremer et al., 2016). Subsequently, the phosphorylated PGC-1 activates the nuclear respiratory factor-1 (NRF-1), which, in turn, regulates the expressions of both mitochondrial and nuclear genes encoding respiratory chain subunits and other proteins that are responsible for mitochondrial biogenesis and function (Wu et al., 1999; Tang et al., 2014). However, these activities have been mainly observed in mammals. Relevant information in fish has been quite limited until now. Recently, some differences have been identified between fish and mammals related to the mitochondrial biogenesis pathway. Accordingly, PGC-1β has been demonstrated to be more effective than PGC-1α when the mitochondrial gene expression was observed, although they have similar capabilities to induce mitochondrial biogenesis in mammals (Bremer et al., 2016). In addition, the mitochondrial function in fish also has been reported to be affected by a large number of factors, including genetics, growth and/or developmental stages, water temperature, diet composition, etc. (LeMoine et al., 2008; Eya et al., 2011, 2012, 2017; Liao et al., 2016). However, information concerning carbohydrate metabolism is still unknown. Considering this, it is of great significance to investigate the potential effects of high-carbohydrate (HC) feeding on the mitochondrial function of fish and characterize the underlying mechanisms. This might facilitate the discovery of effective approaches to improve the mitochondrial function in fish as well as open a new approach to promote its carbohydrate utilization.

Mitochondrial dysfunction is a crucial triggering factor of metabolic diseases such as insulin resistance and diabetes (Kelley et al., 2002; Petersen et al., 2003). At present, accumulating evidence has indicated that the supplementation of mitochondrial nutrients (mt-nutrients) could bring about a series of physiological benefits on mitochondrial structure and function, such as (1) the improvement of mitochondrial membrane structure; (2) the enhancement of mitochondrial enzymes activities by elevating cofactors levels; (3) increase in antioxidant defenses by scavenging excess of free radicals, and so on (Liu and Ames, 2005). Among these mt-nutrients, vitamin B_1_ has attracted considerable attention as it can serve as the essential cofactor to regulate the activity of various mitochondrial enzymes, thereby, improving intracellular mitochondrial function (Depeint et al., 2006). However, vitamin B_1_ is excreted quickly from the body due to its water-soluble characteristic, which can lead to a reduction in its biological functions (Beltramo et al., 2008). Benfotiamine is a lipid-soluble analog of vitamin B_1_ with higher absorption and bioavailability than vitamin B_1_, and is commonly used as a food supplement for the treatment of diabetic complications (Beltramo et al., 2008). It can improve glucose homeostasis by blocking three major pathways associated with hyperglycemic damage: the hexosamine, the advanced glycation end products (AGEs) formation, and the diacylglycerol (DAG)–protein kinase C pathways (Hammes et al., 2003). In addition, benfotiamine administration can remarkably enhance the activity of dehydrogenase enzyme complexes by increasing intracellular thiamine diphosphate (TPP) levels, thereby increasing glucose oxidation in mitochondria (Fraser et al., 2012). Moreover, benfotiamine also has been demonstrated to be able to alleviate the stress caused by the overproduction of superoxide anion in the mitochondrial electron transport chain (Chung et al., 2014). Despite the fact that significant improvements of mitochondrial function have been confirmed in mammals, such information in aquatic animals is extremely scarce. Whether benfotiamine can improve the glucose homeostasis in fish through the enhancement of mitochondrial function still needs to be elucidated.

Blunt snout bream (*Megalobrama amblycephala*) is a commercially important freshwater fish in China (Xu et al., 2016). Due to its herbivorous feeding habits, diets formulated for this fish usually contain large proportions of carbohydrates to reduce the feed cost and maximize the profit. However, severe metabolic burden coupled with the compromised glucose homeostasis is usually observed in this species after the feeding of HC diets (Li et al., 2014). Previously, our study had demonstrated that long-term administration of benfotiamine at 2.85 mg/kg could significantly improve the glucose homeostasis of this species being fed HC diets. However, the underlying mechanisms are still poorly understood. In addition, the growth performance was slightly compromised, suggesting that this dosage might be too high for this species (Xu et al., 2017a). Hence, we speculated that (1) a dose-dependent effect of benfotiamine might exist on the growth performance and intermediary metabolism of fish and (2) benfotiamine might benefit the glucose homeostasis of fish through the promotion of mitochondrial function. Bearing these facts in mind, the present study was conducted to (1) evaluate the effects of different dietary levels of benfotiamine on the growth performance, tissue glycogen and lipid deposition, and levels of plasma metabolites in juvenile blunt snout bream fed an HC diet and (2) investigate its beneficial effects on mitochondrial biogenesis and function in the liver of this species of fish. The findings obtained here might provide us with some new insights into carbohydrate metabolism in fish as well as facilitate the development of nutritional strategies to improve the carbohydrate utilization by aquatic animals.

## Materials and Methods

### Ethics Statement

The care and use of animals in the present study followed the ethical guidelines of the Nanjing Agriculture University in China [permit number: SYXK (Su) 2011-0036]. All experimental procedures involving animals were conducted following the Guidelines for the Care and Use of Laboratory Animals in China.

### Benfotiamine and Diets

Benfotiamine was obtained from Xian Reain Biomedical Company (Xian, China) with a purity of at least 98%. Six isonitrogenous and isolipidic diets were formulated, including a control diet (30% carbohydrate, C), an HC diet (43% carbohydrate), and the HC diet supplemented with different benfotiamine levels [0.7125 (HCB1), 1.425 (HCB2), 2.85 (HCB3), and 5.7 (HCB4) mg/kg, respectively]. Dietary carbohydrate levels were adopted according to our previous studies (Li et al., 2014). Feed formulation and proximate composition of the experimental diets are presented in **Table 1**. Proteins were derived from fish meal, soybean meal, rapeseed meal, and cottonseed meal. Dietary lipids were derived from fish oil and soybean oil. Corn starch was adopted to meet the dietary carbohydrate levels required. Microcrystalline cellulose was included as the filler.

**Table 1:** Formulation and proximate composition of the experimental diets.

	C	HC	HCB1	HCB2	HCB3	HCB4
**Formulation (%)**						
Fish meal	8.00	8.00	8.00	8.00	8.00	8.00
Soybean meal	26.00	26.00	26.00	26.00	26.00	26.00
Rapeseed meal	17.00	17.00	17.00	17.00	17.00	17.00
Cottonseed meal	17.00	17.00	17.00	17.00	17.00	17.00
Fish oil	2.00	2.00	2.00	2.00	2.00	2.00
Soybean oil	2.00	2.00	2.00	2.00	2.00	2.00
Corn starch	12.00	25.00	25.00	25.00	25.00	25.00
Benfotiamine (mg/kg)	0	0	0.7125	1.425	2.85	5.7
Microcrystalline cellulose	13.00	0.00	0.00	0.00	0.00	0.00
Calcium biphosphate	1.80	1.80	1.80	1.80	1.80	1.80
Premix^∗^	1.20	1.20	1.20	1.20	1.20	1.20
**Proximate composition (% *air-dry basis*)**						
Moisture	6.96	6.85	6.92	6.95	6.90	6.87
Crude lipid	5.93	5.71	5.78	5.66	5.77	5.87
Ash	8.46	8.28	8.12	8.23	8.34	8.20
Crude protein	29.82	30.12	30.31	30.03	30.02	30.11
Crude fiber	16.97	6.18	6.29	6.30	6.23	6.28
Nitrogen-free extract^†^	31.86	42.75	42.58	42.83	42.74	42.67
Energy (MJ/kg)	19.09	19.24	19.38	19.31	19.23	19.30


*C, the control diet; HC, the high-carbohydrate diet; HCB1, the HC diet supplemented with 0.7125 mg/kg benfotiamine; HCB2, the HC diet supplemented with 1.425 mg/kg benfotiamine; HCB3, the HC diet supplemented with 2.85 mg/kg benfotiamine; HCB4, the HC diet supplemented with 5.7 mg/kg benfotiamine (the same below). ^∗^Premix supplied the following minerals and/or vitamins (per kg): CuSO_*4*_⋅5H_*2*_O, 2.0 g; FeSO_*4*_⋅7H_*2*_O, 25 g; ZnSO_*4*_⋅7H_*2*_O, 22 g; MnSO_*4*_⋅4H_*2*_O, 7 g; Na_*2*_SeO_*3*_, 0.04g; KI, 0.026 g; CoCl_*2*_⋅6H_*2*_O, 0.1 g; Vitamin A, 900,000 IU; Vitamin D, 200,000 IU; Vitamin E, 4500 mg; Vitamin K_*3*_, 220 mg; Vitamin B_*1*_, 320 mg; Vitamin B_*2*_, 1090 mg; Vitamin B_*5*_, 2000 mg; Vitamin B_*6*_, 500 mg; Vitamin B_*12*_, 1.6 mg; Vitamin C, 5000 mg; Pantothenate, 1000 mg; Folic acid, 165 mg; Choline, 60,000 mg. ^*†*^Calculated by difference (100 - moisture - crude protein - crude lipid - ash - crude fiber).*

### Animals, Experimental Conditions, and Sampling

Juvenile blunt snout bream were purchased from the National Fish Hatchery Station in Yangzhou (Jiangsu, China). Before the experiment, fish were acclimatized to the experimental conditions for 2 weeks, during which they were fed a commercial diet (32% protein, 6% lipids, and 33% carbohydrates) to apparent satiation, manually, three times daily. After acclimatization, 360 fish of similar size (average weight: 45.25 ± 0.34 g) were randomly distributed among 24 indoor tanks (300 L volume) at a number of 15 fish per tank. Fish in each tank were randomly fed with one of the six experimental diets. Each diet was tested in four tanks. Fish were fed to visual satiation thrice daily (07:00, 12:00, and 17:00 h) for 12 weeks. Throughout the experimental period, water temperature averaged 27.4 ± 0.6 °C, pH 7.4–7.5, photoperiod 12: 12 h (dark: light), and dissolved oxygen was maintained above 5.0 mg/L.

After the last meal, all the fish in each tank were fasted for 24 h to empty gut content, and then counted and weighed. Subsequently, 4 fish from each tank were randomly selected and anesthetized with MS-222 (tricaine methanesulfonate; Sigma, United States) at 100 mg/L. Blood was drawn into heparinized tubes as described by Xu et al. (2017b). Liver, muscle, and adipose tissue were removed and immediately frozen in liquid N_2_, and then stored at -80°C until assayed.

### Analysis of Proximate Composition, Plasma and Liver Metabolites, and Tissue Glycogen and Lipid Contents

The proximate composition of diets was determined as follows: dry matter by drying in an oven at 105°C to a constant weight; protein content (nitrogen × 6.25) using the Kjeldahl method after acid digestion (FOSS KT260, Höganäs, Sweden); crude lipid content by ether extraction in a Soxtec System HT (Soxtec System HT6, Tecator, Höganäs, Sweden); ash by incineration in a muffle furnace at 550°C for 4 h; gross energy content by an adiabatic bomb calorimeter (PARR 1281, United States), and crude fiber was determined by the fritted glass crucible method using an automatic analyzer (ANKOM A2000i, United States).

Plasma glucose level was determined using the glucose oxidase method (Asadi et al., 2009). Plasma glycated serum protein (GSP) and AGEs levels were assayed by the method detailed by David and John (1984) and Monnier et al. (1986), respectively. Plasma insulin level was measured using a heterologous radioimmunoassay method using bonito (*Thunnus thynnus*) insulin as the standard and rabbit antibonito insulin as antiserum (Gutierrez et al., 1984). This method has been confirmed in *Cyprinus carpio* (Hertz et al., 1989), which shares the same classification (Cyprinidae family) with *M. amblycephala*. Hepatic contents of adenosine triphosphate (ATP) and AMP were assessed as described by Jaworek et al. (1974) and Lund et al. (1975), respectively. Tissue glycogen and lipid contents were analyzed following the methods detailed by Folch et al. (1957) and Keppler et al. (1974), respectively.

### Analysis of Mitochondrial Respiratory Chain Complex Enzyme Activities

Liver mitochondria isolation was performed using a commercial kit (G006, Nanjing Jiancheng Bioengineering Institute, Nanjing, China). Briefly, after the last meal, fresh liver samples were obtained from another tank with 4 fish, and were then placed immediately in the ice-cold extraction medium consisting of 10 mM KH_2_PO_4_, 250 mM sucrose, and 5 mM ethylenediaminetetraacetic acid. Subsequently, 1 g of liver tissue was homogenized in 10 mL of cold medium. The homogenates were spun down for 10 min at 1,500 *g* in a refrigerated centrifuge. The supernatants were retained in a new centrifuge tube. For the preparation of the mitochondrial fraction, the remaining supernatants were centrifuged by a second spin. The sediment was washed three times with the previously mentioned medium, and was then resuspended in a small volume of medium plus fatty acid-free bovine serum albumin (1 mg/mL). The mitochondrial suspensions were immediately stored at -80°C for subsequent analysis. Mitochondrial protein concentration was determined using the method of Bradford and Dodd (1977). The activities of complex I–III were measured following the methods detailed by Jeejeebhoy (2002). Furthermore, complex IV and V activities were analyzed following the methods of Kirby et al. (2007).

### Western Blot and Quantitative Polymerase Chain Reaction (qPCR) Analysis

Hepatic protein extraction was performed according to our previous study (Xu et al., 2018). Subsequently, protein lysates (20 μg of protein) were separated on a sodium dodecyl sulfate–polyacrylamide gel electrophoresis gel using a Mini-PROTEAN system (BioRad, Spain) for 1–2 h at 100 V. Then, the electroblotted proteins were transferred to a polyvinylidene difluoride (PVDF) membrane (Millipore Corp., Bedford, MA, United States). The specific primary antibodies used were anti-β-actin (BM3873, Boster, China, 1:5000 dilution), anti-AMPKα (#2532, Cell Signaling Technology, United States, 1:2000 dilution), anti-phospho-AMPKα (#2535, Cell Signaling Technology, United States, 1:2000 dilution), anti-PGC-1β (22378-1-AP, Proteintech, United States, 1:1000 dilution), and anti-NRF1 (ab34682, Abcam, Cambridge, MA, United States, 1:1000 dilution). After washing, PVDF membranes were incubated with anti-rabbit (#7074, Cell Signaling Technology, United States, 1:2000 dilution) and anti-rabbit (BA1054, Boster, China, 1:50,000 dilution) secondary antibodies, respectively. Immune complexes were detected by a chemiluminescent substrate (Gel Imagine CHEMI-SMART-3126, France) based on the manufacturer’s instructions, and visualized with a luminescent image analyzer (Fujifilm LAS-3000, Japan). The protein levels were normalized by β-actin, and the intensities of each lane were quantified using the densitometry band analysis tool in Image J 1.44p (U.S. National Institutes of Health, Bethesda, MD, United States).

Total RNA extraction and cDNA synthesis were performed using the liver samples according to our previous studies (Xu et al., 2017a, 2018). Briefly, the quantity and purity of isolated RNA were determined by absorbance measures at 260 and 280 nm, and its integrity was tested by electrophoresis in 1.0% formaldehyde denaturing agarose gels. Specific primers for PGC-1β, NRF-1, Mfn-1, Mfn-2, Opa-1, Drp-1, Fis-1, Mff, and uncoupling protein (UCP)-2 were designed using primer premier 5.0 based on the partial cDNA sequences of the target genes using the transcriptome analysis of blunt snout bream (Gao et al., 2012). The AMPKα-1, AMPKα-2, TFAM, ND-1, CYT B, COX-1, COX-2, and ATP-6 were designed using the published sequences of blunt snout bream (**Table 2**). All primers were synthesized by Invitrogen BioScience & Technology Company (Shanghai, China). Subsequently, the real-time qPCR was run on an ABI 7500 real-time PCR system (Applied Biosystems, Carlsbad, CA, United States) using the SYBR Green II Fluorescence Kit (Takara Bio. Inc., Japan). The fluorescent quantitative PCR reaction solution consisted of 2.00 μL template (equivalent to 500 ng cDNA), 10.00 μL SYBR^®^ premix Ex Taq^TM^ (2×), 0.40 μL Rox Reference Dye II, 0.40 μL PCR Forward Primer (10 μM), 0.40 μL PCR Reverse Primer (10 μM), and 6.80 μL dH_2_O. The qPCR consisted of 40 cycles with the first step of denaturation at 95°C for 30 s and a final extension of 95°C for 5 s and annealing at 60°C for 34 s. All amplicons were initially separated by agarose gel electrophoresis to ensure that they were of the correct size. Finally, the relative transcripts of target mRNA were analyzed by elongation factor 1 α (EF1α) gene using the 2^-ΔΔCT^ method.

**Table 2:** Nucleotide sequences of the primers used to assay gene expressions by real-time PCR.

Target gene	Forward (5′-3′)	Reverse (5′-3′)	Accession numbers or reference
AMPKα-1	AGTTGGACGAGAAGGAG	AGGGCATACAAAATCAC	ARF07712.1
AMPKα-2	ACAGCCCTAAGGCACGATG	TGGGTCGGGTAGTGTTGAG	KX061841
PGC-1β	GTGAGGAACGGGGAGATTG	AGGGGGGTGAACAGGAAAC	Gao et al., 2012
NRF-1	CACAAGCCCTGAGGACTA	ACCTGTATGAGCGAGACG	Gao et al., 2012
TFAM	TCCGAAAGTTAGCAGAGA	ATGAAGATGTTGAAGGCG	KT380498.1
Mfn-1	CTCCAGATGCTCATTCCCT	TTCCTTGGCTTTGGTTGTC	Gao et al., 2012
Mfn-2	ACGCCTCTCCGCTCAAACAC	CTTCCCCAATCCCTGCCACT	Gao et al., 2012
Opa-1	CTTGTTGACTTGCCTGG	TTCATTACGGATGTGCT	Gao et al., 2012
Drp-1	CAGAGGGACTGCGAGGTT	GGCTTGAGCAAAAGGGAA	Gao et al., 2012
Fis-1	ATACAAGCAAAAAAGACGAT	ATACAAAATAAAAAAAGGGG	Gao et al., 2012
Mff	CCCGAGAGAATCGTAGTGG	GGCGTCTTGAGGGACAGTG	Gao et al., 2012
ND-1	CTGACCACTAGCCGCAATA	GGAAGAAGAGGGCGAAGG	NC010341
CYT-b	CATACACTATACCTCCGACAT	TCTACTGAGAAGCCACCT	NC010341
COX-1	CATACTTTACATCCGCAACA	TCCTGTCAATCCACCCAC	NC010341
COX-2	AACCCAGGACCTTACACCC	CCCGCAGATTTCAGAACA	NC010341
ATP-6	TGCTGTGCCACTATGACT	ATTATTGCCACTGCGACT	NC010341
UCP-2	CCAAAGGTCCTGCGAACA	AACCCTACTGCCAATCCC	Gao et al., 2012
EF1α	CTTCTCAGGCTGACTGTGC	CCGCTAGCATTACCCTCC	X77689.1


*AMPKα-1, AMP-activated protein kinase α-1; AMPKα-2, AMP-activated protein kinase α-2; PGC-1β, Peroxisome proliferator activated receptor-γ coactivator-1β; NRF-1, Nuclear respiratory factor-1; TFAM, Mitochondrial transcription factor A; Mfn-1 and 2, Mitofusin-1 and 2; Opa-1, Optic atrophy-1; Drp-1, Dynamin-related protein-1; Fis-1, Fission-1; Mff, Mitochondrial fission factor; ND-1, NADH dehydrogenase-1; CYT-b, Cytochrome-b; COX-1 and 2, Cytochrome c oxidase-1 and 2; ATP-6, ATP synthase-6; UCP-2, uncoupling protein 2; EF1α, Elongation factor 1α.*

### Statistical Analysis

All results are presented as mean ± standard error of the mean (SEM). Before statistical analysis, all data were tested for the normality of distribution and homogeneity of variances among different treatments. Then, data were subjected to one-way ANOVA and Tukey’s multiple tests. Differences were considered significant if a *P*-value of less than 0.05 was obtained. All the statistical analyses were done with SPSS 22.0 for Windows (SPSS Inc, Chicago, IL, United States).

## Results

### Growth Performance

Growth performances of blunt snout bream are shown in **Table 3**. During a 12-week feeding trial, no mortality was observed among all the groups. The final weights (FWs), weight gain rate (WGR), specific growth rate (SGR), relative feed intake (RFI), feed conversion ratio (FCR), and hepatosomatic index (HIS) of fish fed the HC diet were all lower than that of the C group, but significant differences were observed in FW, RFI, and FCR (*P* < 0.05). Additionally, FW, WGR, and SGR all increased significantly (*P* < 0.05) as benfotiamine levels increased from 0 to 1.425 mg/kg, but decreased with further increasing levels. However, both RFI and FCR showed an opposite trend, but no significant difference (*P* > 0.05), with the minimum value observed in fish fed the HCB2 diet. Moreover, benfotiamine supplementation further increased HIS.

**Table 3:** Growth performance of blunt snout bream fed different experimental diets^∗^.

Parameters	Diets					
	
	C	HC	HCB1	HCB2	HCB3	HCB4
IW	46.38 ± 0.63	45.17 ± 0.72	44.81 ± 0.78	47.15 ± 1.11	43.11 ± 1.32	44.79 ± 0.64
FW	167.76 ± 9.54^b^	140.53 ± 9.36^d^	152.62 ± 7.61^c^	185.08 ± 7.75^a^	129.71 ± 8.45^e^	120.30 ± 8.81^e^
WGR †	241.71 ± 6.49^ab^	218.19 ± 9.72^bc^	232.84 ± 7.77^ab^	262.03 ± 11.11^a^	218.10 ± 12.41^bc^	195.20 ± 10.86^c^
SGR §	1.46 ± 0.02^ab^	1.38 ± 0.03^bc^	1.43 ± 0.03^ab^	1.53 ± 0.02^a^	1.38 ± 0.02^bc^	1.29 ± 0.05^c^
RFI ||	2.46 ± 0.03^a^	2.00 ± 0.09^b^	1.96 ± 0.07^b^	1.90 ± 0.07^b^	1.94 ± 0.09^b^	1.99 ± 0.05^b^
FCR ¶	1.89 ± 0.03^a^	1.61 ± 0.08^bc^	1.53 ± 0.05^bc^	1.41 ± 0.04^c^	1.57 ± 0.07^bc^	1.70 ± 0.06^ab^
HSI ††	1.06 ± 0.07	1.15 ± 0.11	1.21 ± 0.15	1.23 ± 0.16	1.21 ± 0.19	1.23 ± 0.12


*IW, Initial weights; FW, Final weights. ^∗^Values are means ± SEM of four replications. Values in the same line with superscripts are significantly different (*P* < 0.05). ^†^Weight gain rate (WGR, %) = (*W*_t_ - *W_*0*_*) × 100 / *W_*0*_*. ^§^Specific growth rate (SGR, % / day) = (*LnW_*t*_* – *LnW_*0*_*) × 100 / *T*, where *W*_*0*_ and *W*_*t*_ are the initial and final body weights, and *T* is the culture period in days. ^||^Relative feed intake (RFI, % body weight d^-*1*^) = Feed intake (g) × 100/[(initial fish weight (g) + final fish weight (g) + dead fish weight (g)) × days reared/2]. ^¶^Feed conversion ratio (FCR) = Feed consumption (g)/fish weight gain (g). ^††^Hepatosomatic index (HSI, %) = liver weight (g) × 100/body weight (g).*

### Plasma Metabolites, Tissue Glycogen and Lipid Contents, and Liver Biochemistry Parameters

**FIGURE 1:**
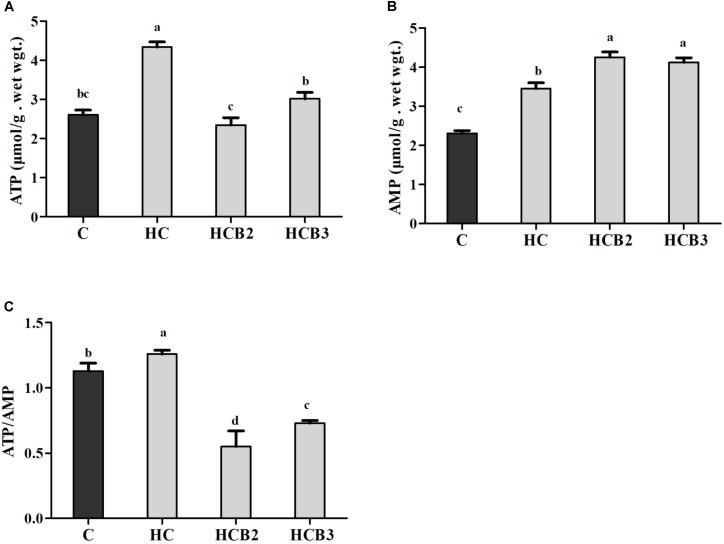
Hepatic ATP **(A)** and AMP **(B)** contents and the ATP/AMP ratio **(C)** of blunt snout bream fed different experimental diets. Each data represent the means ± SEM of four replicates. Bars assigned with different superscripts are significantly different (*P* < 0.05). ATP, adenosine triphosphate; AMP, adenosine monophosphate.

As can be seen from **Table 4** and **Figure 1**, plasma glucose, GSP, AGES and insulin levels, ATP and AMP contents and ATP/AMP ratio as well as liver and muscle tissue glycogen and lipid contents of fish fed the HC diet were all significantly (*P* < 0.05) higher than that of the C group. As for the HC groups, the supplementation of benfotiamine led to a significant (*P* < 0.05) increase of plasma insulin levels, tissue glycogen and lipid contents and AMP contents, whereas the opposite was true for plasma glucose, GSP and AGES levels, and ATP contents and the ATP/AMP ratio.

**Table 4:** Plasma metabolites and tissue glycogen and lipid contents of blunt snout bream fed different experimental diets^∗^.

Parameters	Diets				*P*-value
		
	C	HC	HCB2	HCB3	
Glucose (mmol/L)	3.13 ± 0.02^b^	5.32 ± 0.11^a^	3.31 ± 0.02^b^	3.11 ± 0.13^b^	<0.05
GSP (mmol/L)	14.32 ± 0.12^b^	21.02 ± 0.13^a^	13.27 ± 0.03^bc^	12.76 ± 0.06^bc^	<0.05
AGEs (ng/mL)	3.46 ± 0.03^b^	4.89 ± 0.04^a^	3.31 ± 0.01^b^	3.34 ± 0.02^b^	<0.05
Insulin (ng/mL)	42.31 ± 0.54^c^	73.20 ± 0.82^b^	96.57 ± 0.61^a^	98.19 ± 0.91^a^	<0.05
**Tissue glycogen contents (*μmol glycosyl units/g wet tissue*)**					
Liver	14.31 ± 0.38^c^	23.32 ± 0.30^b^	45.81 ± 0.34^a^	40.44 ± 0.23^a^	<0.05
Muscle	2.46 ± 0.03^c^	3.23 ± 0.02^b^	3.67 ± 0.03^a^	3.81 ± 0.02^a^	<0.05
Adipose tissue	1.42 ± 0.04^b^	1.79 ± 0.15^ab^	2.12 ± 0.07^a^	2.05 ± 0.01^a^	<0.05
**Tissue lipid contents (*percentage of wet weight*)**					
Liver	5.92 ± 0.02^c^	7.54 ± 0.03^b^	8.86 ± 0.08^a^	8.72 ± 0.05^a^	<0.05
Muscle	5.42 ± 0.32^c^	7.22 ± 0.11^b^	7.55 ± 0.21^b^	9.13 ± 0.45^a^	<0.05
Adipose tissue	76.03 ± 0.41^ab^	81.21 ± 0.15^ab^	85.23 ± 0.20^a^	84.58 ± 0.19^a^	<0.05


*GSP, glycated serum protein; AGEs, advanced glycation end products. ^∗^Values are means ± SEM of four replications. Values in the same line with superscripts are significantly different (*P* < 0.05).*

### Hepatic Protein Expressions of AMPKα, PGC-1β, and NRF-1

**FIGURE 2:**
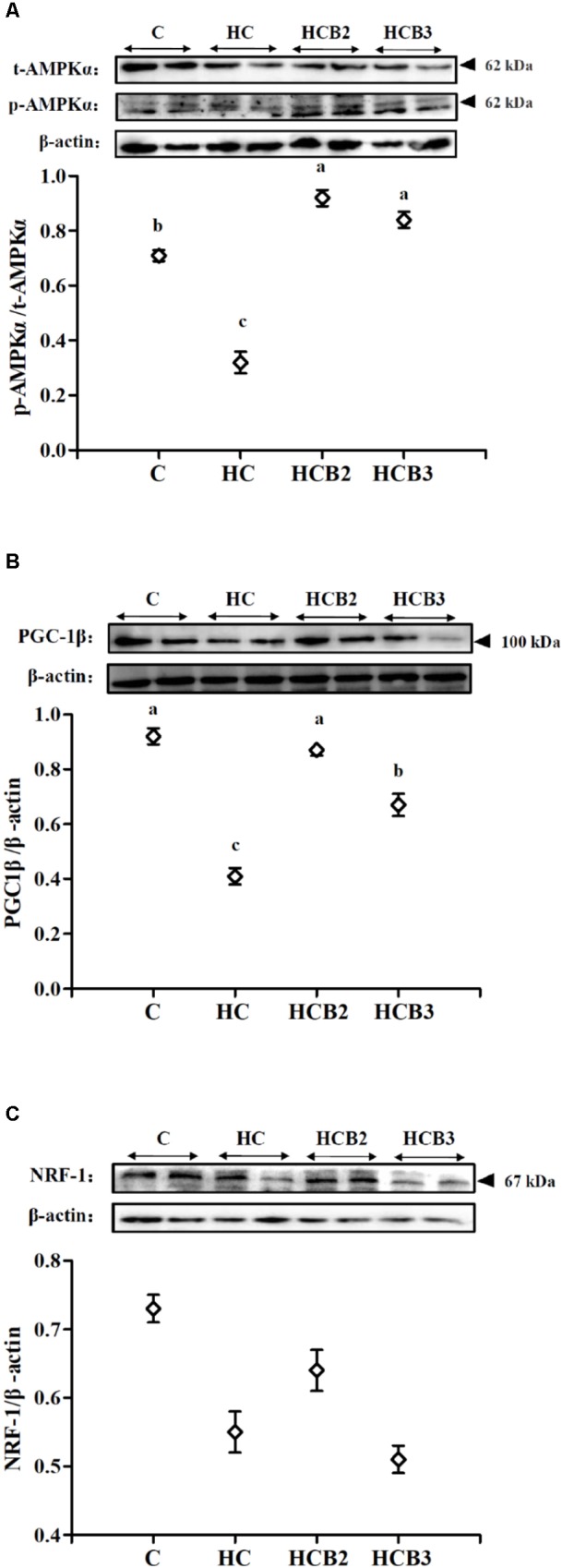
The protein expressions of AMPKα **(A)**, PGC-1β **(B)**, and NRF-1 **(C)** in the liver of blunt snout bream fed different experimental diets. Two blots were presented for each treatment. Gels were loaded with 20 μg total protein per lane. Each data represent the means ± SEM of four biological replicates. Bars assigned with different superscripts are significantly different (*P* < 0.05).

As can be seen from **Figure 2**, fish fed the HC diet had significantly (*P* < 0.05) lower *p*-AMPKα/*t*-AMPKα ratio and PGC-1β protein expressions than that of the C group. As for the HC groups, the supplementation of benfotiamine significantly increased (*P* < 0.05) the *p*-AMPKα/*t*-AMPKα ratio and PGC-1β protein expression, while little difference (*P* > 0.05) was observed in the NRF-1 content.

### Hepatic Transcriptions of the Genes Involved in Mitochondrial Biogenesis

**FIGURE 3:**
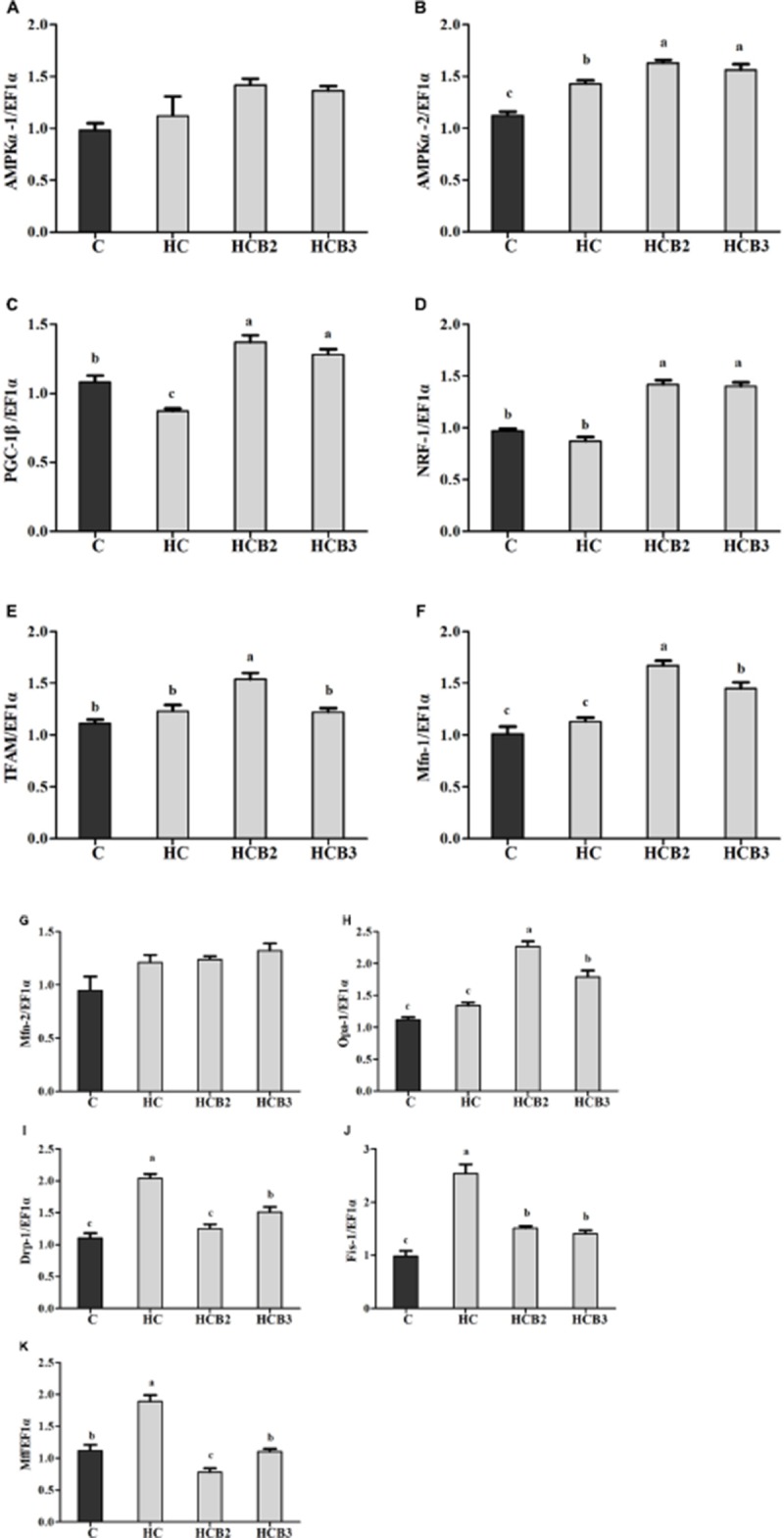
The relative transcriptions of mitochondrial biogenesis-related genes in the liver of blunt snout bream fed different diets. The transcriptional levels of AMP-activated protein kinase α-1 and 2 (AMPKα-1 and 2) **(A,B)**, Peroxisome proliferator activated receptor-γ coactivator-1β (PGC-1β) **(C)**, Nuclear respiratory factor-1 (NRF-1) **(D)**, Mitochondrial transcription factor A (TFAM) **(E)**, Mitofusin-1 and 2 (Mfn-1 and 2) **(F,G)**, Optic atrophy-1 (Opa-1) **(H)**, Dynamin-related protein-1 (Drp-1) **(I)**, Fission-1 (Fis-1) **(J)** and Mitochondrial fission factor (Mff) **(K)** were all evaluated using real-time RT-PCR. Expression levels were normalized to EF1α-expressed transcripts and are presented as fold-change against the control (C) group set to 1. Each data represent the means ± SEM of four replicates. Bars assigned with different superscripts are significantly different (*P* < 0.05).

As can be seen from **Figure 3**, no statistical difference (*P* > 0.05) was observed in the transcriptions of AMPKα-1 and Mfn-2 among all the treatments. Fish fed the HC diet had significantly (*P* < 0.05) higher transcriptional levels of AMPKα-2, Drp-1, Fis-1, and Mff than that of the C group, whereas the opposite was true for PGC-1β expression. As for the HC groups, the supplementation of benfotiamine significantly increased (*P* < 0.05) the transcriptional levels of AMPKα-2, PGC-1β, NRF-1, TFAM, Mfn-1, and Opa-1 (with the maximum values all observed in fish fed the HCB2 diet), whereas the opposite was true for Drp-1, Fis-1, and Mff.

### Hepatic Activities of the Enzymes Involved in Mitochondrial Function

**FIGURE 4:**
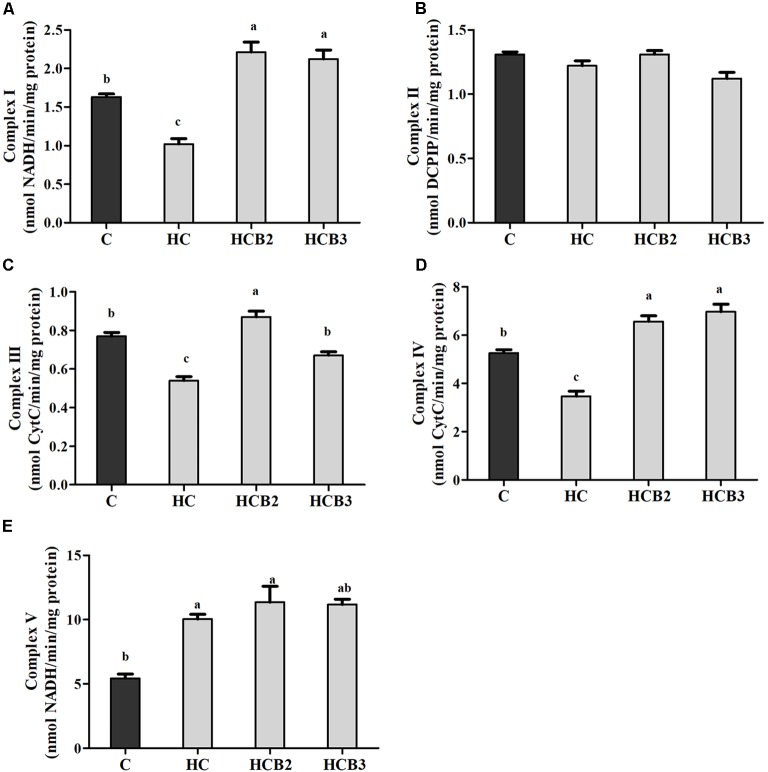
The activities of mitochondrial respiratory chain complexes (Complex I **(A)**, II **(B)**, III **(C)**, IV **(D)** and V **(E)**) in the liver of blunt snout bream fed different experimental diets. Each data represent the means ± SEM of four replicates. Bars assigned with different superscripts are significantly different (*P* < 0.05). Complex I: NADH–ubiquinone oxidoreductase; Complex II: Succinate–ubiquinone oxidoreductase; Complex III: Ubiquinone–ferricytochrome-c oxidoreductase; Complex IV: Cytochrome c oxidase; Complex V: F1F0–ATP synthase. They were expressed as nanomolars per minute per milligram protein.

As can be seen from **Figure 4**, no significant difference (*P* > 0.05) was observed in the activities of complex II among all the treatments. Fish fed the HC diet had significantly (*P* < 0.05) lower activities of complex I, III, and IV than that of the C group, whereas the opposite was true for complex V activities. As for the HC groups, the supplementation of benfotiamine significantly increased (*P* < 0.05) the activities of complexes I–V, with the maximum values all observed in fish fed the HCB2 diet.

### Hepatic Transcriptions of the Enzymes Involved in Mitochondrial Function

**FIGURE 5:**
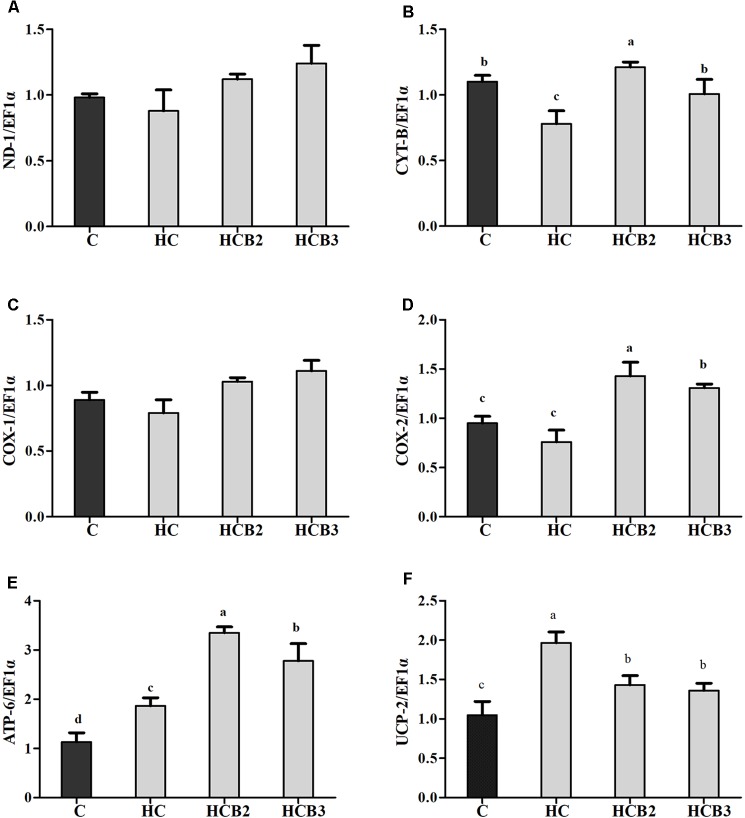
The relative transcriptions of mitochondrial function-related genes in the liver of blunt snout bream fed different diets. The transcriptional levels of NADH dehydrogenase-1 (ND-1) **(A)**, Cytochrome-b (CYT-b) **(B)**, Cytochrome c oxidase-1 and 2 (COX-1 and 2) **(C,D)**, ATP synthase-6 (ATP-6) **(E)**, and Uncoupling protein 2 (UCP-2) **(F)** were all evaluated using real-time RT-PCR. Expression levels were normalized to EF1α-expressed transcripts and are presented as fold-change against the control (C) group set to 1. Each data represent the means ± SEM of four replicates. Bars assigned with different superscripts are significantly different (*P* < 0.05).

As can be seen from **Figure 5**, no significant difference (*P* > 0.05) was observed in the transcriptions of ND-1 and COX-1 among all the treatments. Fish fed the HC diet had significantly (*P* < 0.05) lower transcriptional level of CYT-b than that of the C group, whereas the opposite was true for ATP-6 and UCP-2 expression. As for the HC groups, the supplementation of benfotiamine significantly increased (*P* < 0.05) the mRNA levels of CYT-b, COX-2, and ATP-6, with the maximum values all observed in fish fed the HCB2 diet.

## Discussion

In the present study, HC intake led to a decreased value of FW, WGR, SGR, RFI, and FCR in blunt snout bream, but HSI is on the opposite trend. This result is consistent with the fact that high-energy diets easily reduce feed palatability, and usually accelerate animal satiety, thereby, leading to low feed consumption (Ali and Jauncey, 2004). Moreover, high dietary carbohydrates usually results in persistent hyperglycemia, which is regarded as a physiological stress response, retarding the growth of fish (Hemre et al., 2002). As for the HIS, it showed similar trends as the results of liver glycogen and lipid contents although no statistical difference was observed, which is similar to the results of our previous study (Xu et al., 2017a). This may be ascribed to the fact that as a herbivorous species, blunt snout bream has higher glucose tolerance, which can quickly remove excessive glycogen and lipid from the liver. In addition, FW, WGR, and SGR were significantly improved by benfotiamine supplementation with increasing level up to 1.425 mg/kg, while the opposite was observed in RFI and FCR. These findings suggested a beneficial effect of benfotiamine on the growth performance of fish fed the HC diet at a suitable dosage (namely 1.425 mg/kg). According to a previous study, adequate thiamine (the analog of benfotiamine) levels could promote the activities of intestinal digestive and brush border enzymes of fish, thus improving nutrients absorption and feed efficiency, and this might promote the growth performance (Huang et al., 2011). In addition, benfotiamine administration could also diminish the hyperglycemic damage in mammals through the inhibition of AGEs formation and other metabolic pathways (Hammes et al., 2003). It is possible that a similar mechanism also exists in fish. However, further studies are warranted to elucidate these facts. Together, these effects might be beneficial to the glucose homeostasis of fish fed carbohydrate-enriched diets, thereby, improving the carbohydrate utilization by fish. In addition, benfotiamine supplementation further increased the HIS, but with no significant difference, indicating benfotiamine can accelerate the removal of excessive nutrients deposited in the liver. This result is also beneficial for enhancing liver metabolic function, since the excessive accumulation of nutrients will result in histological and pathological damage of the liver (Hilton and Hodson, 1983; Caballero et al., 2004). Meanwhile, the HCB3 diet led to a decrease of FW, WGR, RFI, and FCR compared with the HC group. This showed similar trends to the results of our previous work (Xu et al., 2017a), although no statistical difference was observed here. Generally, this difference may be ascribed to the following differences between the previous and the current study, which might include the initial body size of fish, the stocking density, the feed consumption, the coefficient variation of the data collected within each treatment, the water temperature during the feeding trial, and so on. Furthermore, both the WGR and SGR decreased significantly with further increasing benfotiamine levels, indicating that the overdose of this substance could impair the growth performance of fish. The physiological basis for such growth retardation is still absent in fish until now. To further characterize the underlying mechanisms, molecular investigations were conducted in certain groups (namely the C, HC, HCB2, and HCB3).

In this study, fish fed the HC diet exhibited relatively high values of plasma glucose, GSP, AGES, and insulin as well as tissue glycogen and lipid compared with the control group. This suggested that high dietary carbohydrate intake induced a hyperglycemic state of fish. It is generally acknowledged that high glucose levels induced by HC intake usually stimulate the synthesis and release of insulin, thus accelerating glucose disposal in peripheral insulin target tissues by enhancing glycolysis and glycogenesis (Polakof et al., 2012; Kamalam et al., 2017). Meanwhile, the elevated intracellular glucose levels also enhanced the Maillard reaction, thereby, increasing AGES levels (Beltramo et al., 2008). In addition, the results of GSP, an accurate and easily detectable intermediate marker of glycemia, in this study, further supported the afore-mentioned facts (Selvaraj et al., 2008). The enhanced tissue glycogen and lipid deposition is also justifiable, since HC intake generally promotes the glycogenesis and lipogenesis of fish (Enes et al., 2009). In addition, dietary supplementation of benfotiamine further increased plasma insulin levels and tissue glycogen and lipid content, whereas the opposite was true for plasma glucose, GSP, and AGES levels. These results suggested that benfotiamine could improve the glucose homeostasis of blunt snout bream fed a HC diet. This may be due to the following facts: (1) benfotiamine could stimulate insulin synthesis by improving the function of pancreatic β-cells, thus resulting in low plasma glucose and GSP levels (Rathanaswami and Pourany, 1991); (2) benfotiamine could accelerate the removal of intracellular glycerhaldeyde-3-phosphate (G3P), thereby decreasing the formation of AGES (Beltramo et al., 2008); and (3) benfotiamine could enhance the pentose phosphate pathway and fatty acid synthesis, thus increasing lipid accumulation (Berrone et al., 2006; Beltramo et al., 2008). However, these are the cases for mammals. Whether fish show a similar mechanism is still uncertain, and this warrants further study. Meanwhile, the elevated insulin levels usually enhance the activities of glycogen synthase (GSase) and the dephosphorylation of glycogen phosphorylase (GPase), which might promote glycogen storage in tissues in fish (Moon, 2001).

Generally, ATP is the major energy “currency” in cells, and the ATP/AMP ratio is a sensitive indicator of the alterations of the energy status (Yoshida et al., 2012). In the present study, the hepatic ATP and AMP contents and ATP/AMP ratio of fish fed the HC diet were all significantly higher than that of the control group. The most plausible explanation would be that high dietary carbohydrate intake usually elevates the energy state of cells, thus increasing ATP contents (Sathanoori et al., 2015). The excessive ATP was hydrolyzed consequently, thus leading to the increased AMP content. Moreover, dietary benfotiamine supplementation led to the increased AMP contents, while the opposite was true for ATP contents and the ATP/AMP ratio. This indicated that benfotiamine could modify the intracellular energy state of fish. According to a previous study, as a thiamin analog, benfotiamine could accelerate ATP hydrolysis via the following reaction: thiamine + ATP →thiamine diphosphate (ThDP) + AMP, thereby decreasing the ATP/AMP ratio (Makarchikov, 2009). In addition, the decreased ATP/AMP ratio by benfotiamine administration is regarded as a positive signal for glucose homeostasis, since it could activate some specific energy sensors (such as AMPK), thereby coordinating the glycolipid metabolism of fish (Rutter and Leclerc, 2009).

Current studies have demonstrated that mitochondrial fusion and fission processes play a prominent role in the modulation of mitochondrial biogenesis and function (Rossignol and Karbowski, 2009; Su et al., 2009). Additionally, mitochondrial fusion and fission enzymes have also attracted considerable attention due to their critical roles in controlling the dynamic events mentioned earlier (Su et al., 2009). However, such information in fish is still barely understood. In this study, hepatic transcriptions of AMPKα-1, Drp-1, Fis-1, and Mff were all upregulated by the intake of carbohydrate-rich diets compared with the control group, whereas the opposite was true for PGC-1β and NRF-1 transcriptions as well as PGC-1β and NRF-1 protein expressions and the *p*-AMPKα/*t*-AMPKα ratio. According to previous studies, the increased intracellular ATP contents by HC intake usually inhibit the activity of AMPK in fish (Polakof et al., 2011a,b). Subsequently, the inactive AMPK could weaken mitochondrial biogenesis signals by reducing the activity of the PGC-1/NRF-1 pathway, resulting in a decrease of mitochondrial content in cells (Yu and Yang, 2010). Meanwhile, excessive carbohydrate intake also amplifies mitochondrial oxidative stress by increasing the generation of intracellular reactive oxygen species (ROS), thereby accelerating mitochondrial fission characterized by the upregulation of related genes (namely Drp-1, Fis-1, and Mff) (Zhuang et al., 2017). In addition, dietary supplementation of benfotiamine at 1.425 mg/kg significantly upregulated the transcriptions of AMPKα-2, PGC-1β, NRF-1, TFAM, Mfn-1, and Opa-1, the protein contents of PGC-1β as well as the *p*-AMPKα/*t*-AMPKα ratio, whereas the opposite was true for Drp-1, Fis-1, and Mff. These results indicated that a long-term administration of benfotiamine at the appropriate dosages effectively enhanced the mitochondrial biosynthesis of fish fed the HC diet. According to a previous study, benfotiamine could decrease the ATP/AMP ratio by accelerating ATP hydrolysis, thereby increasing the activity of AMPK (Makarchikov, 2009). Once activated, AMPK conveys its signals to induce mitochondrial biogenesis via targeting the PGC-1β/NRF-1 pathway that regulates mtDNA replication and expression (Wu et al., 1999). Furthermore, these results were further supported by the fact that AMPK activation can prevent high glucose-induced mitochondrial fission by inhibiting the activity of the proteins involved in mitochondrial fission (such as Drp-1 and Mff), thus coordinating this organelle shape and function (Wikstrom et al., 2013; Ducommun et al., 2015; Craig et al., 2017). It should be stated here that this information was mainly derived from mammals. The underlying mechanisms in fish still need further in-depth studies. Nevertheless, the enhanced mitochondrial biogenesis by benfotiamine may be helpful for the maintenance of glucose homeostasis in this fish, thus improving its carbohydrate utilization.

Mitochondrion is the main site of energy production in cells, and responsible for the production of ATP for the basic activities of life (Wang et al., 2017). Generally, ATP synthesis in mitochondria depends on the oxidative phosphorylation (OXPHOS) by means of an enzyme pathway consisting of five multisubunit enzyme complexes located within the mitochondrial inner membrane (Vedel et al., 1999). Therefore, investigations of these enzyme complexes will facilitate our understanding of the mitochondrial function. In the present study, the hepatic complexes I–IV activities of fish fed the HC diet were all significantly lower than that of the control group, whereas the opposite was true for the activities of complex V. These results might be attributed to the fact that the oxidative stress induced by HC intake would cause mitochondrial damage, then leading to the decreases in mitochondrial respiratory enzyme activities (Li et al., 2001; Lin et al., 2009). As for the increased complex V activities, it might be a result of the increased energy intake by fish due to HC feeding, which is in line with the trend of ATP content. This is due to the fact that complex V (ATP synthase) is the last rate-limiting enzyme in ATP synthesis, driving the phosphorylation of ADP to ATP (Vedel et al., 1999; Tang et al., 2014). These trends were supported by the fact that high energy consumption generally causes the mitochondria to increase oxidative phosphorylation rate and ATP production (Jonathan et al., 2010), thereby enhancing the activities of complex V (namely the ATP synthase) (Hatefi, 1985). Similar results were also reported in rainbow trout (*Oncorhynchus mykiss*) (Eya et al., 2017) and channel catfish (*Ictalurus punctatus*) (Jonathan et al., 2010). Moreover, dietary benfotiamine supplementation led to the increased complexes I–V activities. This indicated that benfotiamine could enhance mitochondrial function of fish. According to a previous study, benfotiamine could prevent mitochondria from oxidative stress by scavenging free radicals and inhibiting oxidants production, thus enhancing the mitochondrial respiratory enzyme activities (Liu and Ames, 2005). Furthermore, a previous study investigating the effects of the B vitamin family on mitochondrial function further supported this by demonstrating that vitamin B_1_ is an important cofactor for various mitochondrial enzyme complexes (Depeint et al., 2006). Meanwhile, we further evaluated the mitochondrial function by detecting the transcriptional levels of related genes. The transcription of CYT-b in liver decreased remarkably with increasing dietary carbohydrate levels, whereas the opposite was true for ATP-6 expression. In addition, the transcripts of ND-1, COX-1, and COX-2 were all downregulated by the HC diet, although no significance was detected. These results were in accordance with that of the mitochondrial complexes activities, suggesting that a long-term intake of the HC diet could result in an impaired mitochondrial function in this fish. This was supported by the fact that the decreased expressions of ND-1, CYT-b, COX-1, and COX-2 might affect the assembly of mitochondrial complexes, then reducing the catalytic ability of OXPHOS (Eya et al., 2012; Wang et al., 2017). Furthermore, the excessive production of ROS resulting due to the HC intake would also accelerate mitochondrial fission, which might, in turn, depress the transcriptions of these genes (Qian et al., 2005; Zhuang et al., 2017). The upregulated ATP-6 expression might be attributed to the increased energy intake caused by HC diets, which could increase the oxidative phosphorylation rate and ATP production in mitochondria, then enhancing its transcriptional level (Jonathan et al., 2010). Here, an increased UCP-2 expression was also found in the HC group, suggesting that HC intake induced the uncoupling reaction in the respiratory chain. This phenomenon seems to be the opposite of ATP content, but it is not unique to our system. Infact, previous studies have confirmed that high-sucrose diet intake could also accelerate the phosphorylation of ADP to produce ATP by stimulating the coupling reaction in liver mitochondria despite the overexpression of UCP (Ruiz-Ramírez et al., 2011). These results may suggest that the expression of UCPs and mitochondrial respiration are not completely interconnected (Herlein et al., 2009; Ruiz-Ramírez et al., 2011). However, the underlying mechanisms involving these aspects mentioned previously are still unknown. Meanwhile, dietary supplementation of benfotiamine at 1.425 mg/kg resulted in a remarkable increase of the transcriptions of CYT-b, COX-2, and ATP-6. This result indicated an enhanced mitochondrial function in the liver of blunt snout bream due to the administration of benfotiamine at suitable dosages. This was further supported by the fact that benfotiamine could scavenge excess free radicals as well as inhibit superoxide anion production, thus alleviating the functional damage of mitochondria caused by high glucose-induced oxidative stress (Liu and Ames, 2005). The studies afore-mentioned are mainly focused on mammals. The exact mechanisms in fish still warrant further in-depth studies.

In summary, the findings of the present study indicated that dietary supplementation of benfotiamine could improve the growth performance and mitochondrial biogenesis and function of *M. amblycephala* fed HC diets, through the activation of the AMPK/PGC-1β/NRF-1 pathway, the upregulation of the fusion-associated genes, and the enhancement of mitochondrial enzyme complexes activities as well as their transcriptions. The best value of growth performance and mitochondrial biogenesis and function were all observed in fish offered 1.425 mg/kg benfotiamine.

## Author Contributions

X-FL, CX, and W-BL conceived and designed the experiments. CX and D-DZ analyzed the data. X-FL, CX, H-JS, and LZ performed the experiments and contributed reagents, materials, and analysis tools. CX and X-FL wrote the paper. All authors read and approved the final version of the manuscript.

## Conflict of Interest Statement

The authors declare that the research was conducted in the absence of any commercial or financial relationships that could be construed as a potential conflict of interest.
